# Medicalization of global health 1: has the global health agenda become too medicalized?

**DOI:** 10.3402/gha.v7.23998

**Published:** 2014-05-16

**Authors:** Jocalyn Clark

**Affiliations:** 1 Communications & Development Unit, icddr,b, Dhaka, Bangladesh; 2 Department of Medicine, University of Toronto, Toronto, Canada

**Keywords:** Global health, medicalization, sociology, development

## Abstract

Medicalization analyses have roots in sociology and have critical usefulness for understanding contemporary health issues including the ‘post-2015 global health agenda’. Medicalization is more complex than just ‘disease mongering’ – it is a process and not only an outcome; has both positive and negative elements; can be partial rather than complete; and is often sought or challenged by patients or others in the health field. It is understood to be expanding rather than contracting, plays out at the level of interaction or of definitions and agenda-setting, and is said to be largely harmful and costly to individuals and societies. Medicalization of global health issues would overemphasise the role of health care to health; define and frame issues in relation to disease, treatment strategies, and individual behaviour; promote the role of medical professionals and models of care; find support in industry or other advocates of technologies and pharmaceuticals; and discount social contexts, causes, and solutions. In subsequent articles, three case studies are explored, which critically examine predominant issues on the global health agenda: global mental health, non-communicable disease, and universal health coverage. A medicalization lens helps uncover areas where the global health agenda and its framing of problems are shifted toward medical and technical solutions, neglecting necessary social, community, or political action.

Medicalization is a process by which human problems come to be defined and treated as medical problems. It involves the application of a biomedical model that sees health as freedom from disease and is characterised by reductionism, individualism, and a bias toward the technological (Box 1). Critical examinations of medicalization and its limitations for understanding health owe much to a long tradition of study by sociologists who have uncovered the ways that such varied conditions as addiction, childbirth, infant feeding, sadness, erectile dysfunction, and death have become medical issues to be treated (1–6). Traditionally, medicalization was seen to be fuelled by the medical profession and doctors, whose cultural authority and power allowed them to grow medical jurisdiction over people's health and society, resulting in what Illich described as ‘medical imperialism’ (7). More recent analyses affirm the medicalization of many aspects of life and identify the changing drivers, particularly in the United States and Europe: pharmaceutical industry interests, the rise of biotechnology and genomics, and health systems like ‘managed care’ that limit provision or choice, all of which wield increasing power for defining ‘legitimate’ health problems (8, 9). Some commentators have drawn upon insights from medicalization analyses to identify overdiagnosis and ‘disease mongering’, particularly by the pharmaceutical industry intent on creating new disease categories to sell drugs (10).

Contemporary sociological analyses recognise complexity in medicalization: the fact that medicalization is a process not just an outcome, it can be both positive and negative, partial rather than complete, and can be sought by patients, doctors or other actors in the health field, as well as be resisted or challenged (8, 11, 12). Nevertheless, the consequences of medicalization are largely seen to be negative for both individuals and societies (7–13): pathologising normal behaviour, disempowering individuals when subject to control by medical professionals or models of care, decontextualising experience, and depoliticising social problems. As Parens argues, ‘insofar as medicine focuses on changing individuals’ bodies to reduce suffering, its increasing influence steals attention and resources away from changing the social structures and expectations that can produce such suffering in the first place’ (13). And medicalization incurs substantial costs for health care treatments and side effects: Conrad and colleagues recently estimated the costs of medicalization of 12 conditions to be $77 billion per year in the United States alone (14).

Medicalization can occur at multiple levels (12) – for example, at the level of interaction where individual patients or doctors seek a medical label or apply a medical solution to a problem, or at a higher level where definitions, priorities, and agendas are set and recommended strategies and resource allocations determined. Medicalization analyses are particularly valuable at this higher level (8), helping uncover how certain issues in the health field get defined, how others are excluded, how the solutions to problems are constructed, and what agendas are promoted. This is where it comes in as a critical tool for global health.

While medicalization analyses (almost exclusively conducted in the Western context) have identified problems with ‘creating’ diseases and patients, overlooking social causes for ill health, and promoting pharmaceutical treatments rather than broader political change, there is a need to extend these analyses to explore the medicalization of the global health agenda. As other scholars have affirmed, it is of critical importance to examine the relationship of medicine to emerging priorities and consensus on global health. For example, some have highlighted that biomedical advances alone do not alleviate health problems, and nor have technological solutions or the ability to ‘cure’ or treat been responsible historically for public health gains (15–17). More critical scholars, like Navarro, Coburn, and other social scientists (18–21), have highlighted the political determinants of health, demonstrating how the concentration of power, ideology (such as neoliberalism), and other dimensions of politics can have an enormous impact on the health indicators and inequalities of societies; examples include structural adjustment and poverty reduction strategies, the decentralisation of health systems, and other market-driven technical solutions that grew out of neoliberal economic policies and are criticised for distorting and negatively impacting global health and development. More contemporary work by groups such as Global Health Watch (www.ghwatch.org), Go4Health (22), and *The Lancet* – University of Oslo Commission on Global Governance for Health (23) build on this recognition of the political context of health to offer critical analyses of the current global health policy agenda and of the limits of medicine.

***Box 1.*** Forms of medicalization.

Medicalization occurs when the biomedical model – the modern, dominant form of practice by Western health care professionals – is applied to the understanding of a phenomenon. A biomedical model sees health as freedom from disease, and is characterised by reductionism, individualism, and a bias toward the technological.

Reductionism

- Ignores or excludes context and reduces explanations for problems to the physical realm, overlooking social, cultural, psychological, or environmental factors that contribute to or influence why a phenomenon occurs (1).
- Depends upon normative ways of thinking that exclude complexity, relativity, and multiplicity of experiences of health.
- Seeks causes and solutions in biology rather than in social or political forces, depending upon tests, images, and diagnoses to treat deficiencies and restore health (2).
- *For example*, in the classic case of the medicalization of alcoholism that saw the previously deviant problem go from ‘badness to sickness’, medical responses framed the problem not as moral weakness or a social problem but as a failure of biochemistry or genetics, and developed medical strategies to treat the brain-based condition (3).

Individualism

- Places the responsibility and sometimes blame for problems with an individual rather than with structures that shape or determine that individual's behaviour or experience.
- *For example*, Scott's case of the medicalization of shyness shows how a biomedical approach to shyness ‘reinforces the belief that this is a problem of individual minds rather than a reflection of social norms and values’ that expect assertiveness, self-confidence, and gregarious participation in life; any deviation can and should be ‘treated’ (4). *Another example*: Analyses of the medicalization of attention deficit hyperactivity disorder (ADHD) highlight medical values that cast disruptive, inattentive behaviour of children into a mental or brain disorder with treatments: the efficiency of drug therapy, the interests and profits of drug companies, and control of children's behaviour (of interest to classmates, teachers, and parents) (5).
- Can deflect attention away from the government's role or responsibility in addressing a problem and from actions like industry marketing behaviour. *For example*, in the medicalization of child malnutrition, Global Health Watch criticises the UNICEF approach that focuses on supplementation with ready-to-use foods and ignores the economic constraints, poverty, barriers to breastfeeding, food pricing, and trade policies that result in mothers and children not receiving adequate and sustained nutrition (6).

Technological bias

- It has its roots in the biomedical precept of the body as machine, inspiring a focus on the curative elements of medicine rather than preventative actions such as changes in the environment (1).
- Technological imperative favours drug, device, or other medical technologies or other ‘magic bullets’ to treat problems.
- *For example*, analyses of the medicalization of death show a natural state that has become more and more under the purview of medicine and biotechnology, which now regulates the circumstances and exact moment of death (5). This control as been fought by proponents of a more natural death, but across geography and class the conditions of death and dying continue to be medicalized (5).

As Benatar states, the medicalization of health produces ‘too simplistic a view of making more modern medical treatments available to more people’ (16). In the global health context, medicalizing priority problems and solutions may prove detrimental for how the world responds and resources actions designed to alleviate poor health and poverty, redress inequities, and save lives. Examining whether the global health agenda is being defined and fashioned in this way is particularly relevant in light of the striking rise of global health.

## The rise of global health

The field of global health is growing enormously in size and profile. What was once a marginal field within medicine and the health sciences is now an abundant area of research, education, and policy, and has become ‘fashionable’ among students and practitioners (24). Partly this is due to increased visibility of the levels of preventable mortality and morbidity around the world, especially the disproportionate burden of disease borne by developing countries, recognition that a billion of the poorest people in the world live in middle-income rather than poor countries (25), and the fact that the global epidemiological transition is creating a double burden of infectious and chronic disease threats, highlighting how social conditions of poverty and industrialisation combine (26). Governments and international agencies have recognised social and collective responsibility for improving the world's health, as well as the links between economic development and health, and as such the need to invest effort into global health. High profile international funding initiatives like GAVI and the Global Fund to Fight AIDS, Tuberculosis and Malaria demonstrated multi-lateral commitments to fighting common problems, and the growth of philanthropic activity, especially the enormous endowment of the Bill & Melinda Gates Foundation, have put global health concerns on centre stage. Between 1990 and 2008 donor funding to global health dramatically increased from US$5.6 billion to 21.8 billion (27) and, despite the fiscal crisis of 2008, global health financing expanded to $26.9 billion in 2010 (28).

While the breadth of global health problems concerns multidisciplinary fields such as law, business, and public policy, the profile of global health appears most elevated by its expanded presence within medical institutions including professional curriculum and medical journals. In 2008, nearly half of all American medical schools had some activity dedicated to global health (29), and in 2013 the Consortium of Universities for Global Health listed over 100 university global health programmes in North America; similar up-trends are occurring in Europe and worldwide (30). Global health research and debate now regularly appear in the highest impact weekly medical journals, including *BMJ, The Lancet*, and *PLOS Medicine*, which also often now editorialise and campaign on matters of global health. More recently even the clinically inclined US-based medical journals *JAMA* and *NEJM* have developed sections on global health, and several ‘specialist’ journals devoted to global health research and practice (e.g. *Global Health Action, Journal of Global Health, and Lancet Global Health*) have emerged to address growing interest and the needs of a diverse global health community.

## The global health agenda

Similarly, global health now occupies a central place in the world's development discussions, including a notable presence at the 2013 United Nations General Assembly (31), with the recognition that a healthy population is an economically productive one. Addressing top development issues like poverty, education, and environmental sustainability is seen to require good health, and governments’ foreign policy, security, and humanitarian interests now link health with development. The commitment of the UN's 189 member states to the Millennium Declaration led to the Millennium Development Goals (MDG) programme, which defined eight development targets, of which three were explicitly named as health targets (reduce maternal and child deaths, and cut the rate of HIV and malarial infections), and has galvanised international attention, effort, and investment.

With the MDG end-date approaching, attention is focused on the next round of development goals. ‘Post-2015’ architects and campaigners look to learn from the lessons of the MDG programme – what succeeded, what failed, and how to optimise the place of health within the development agenda for the next two decades. A plethora of research, commentary, opinion, and reports of various consultations and high-level meetings dominate global health conversations and journals; leading issues include universal health coverage and non-communicable diseases including mental health. Amidst the flurry of activity to develop and influence the post-2015 development agenda, it seems clear that competition is fierce: contending priorities must capture often elusive political momentum and commitment, and must do so in light of global fiscal restraint and increasing rivalry for attention and funding on the international stage (28).

## What kind of problem is global health?

So while health is considered a key ingredient for development, how is ‘health’ defined? This question is critically important, particularly in light of the predominance global health has on the post-2015 development agenda discussions, and for what it will imply for actions and policy responses. One major on-going tension has been the relative recognition of the social determinants of health. Social determinants include income, education, employment, housing, nutrition, and other individual and social factors that relate to ‘the conditions in which people are born, grow, live, work, and age, and the inequities in power, money, and resources that give rise to them’ (32). Their importance to health has been recognised for decades and enshrined in international declarations such as Alma Ata (33), and much recent commentary (34–37) has stressed the importance of including social determinants in the new health agenda post-2015 and critiqued the MDG programme for failing to sufficiently address these underlying causes of ill health and equity. Others broaden the lens further and argue for attention to the wider political context and systems, which Birn says are ‘the causes of the causes of the causes’ (15). Similarly, Navarro and others argue that no consideration of the determinants of health is complete without examining the politics and power relations of the system in which priorities emerge (18–21). Health, under this global political economy approach, is a production of economic and trade policies, governance, human rights protection, and other societal determinants (15, 16). Health care, dominated by biomedicine, is but one of multiple determinants of health.

Still, a medical definition of global health has been evident in a variety of international initiatives: the multi-lateral partnership of the Global Fund whose main mission is to improve access to drugs for AIDS, malaria, and tuberculosis rather than the living conditions that risk and mitigate infection (16); the technological focus and priorities of the Gates Foundation (38); and the reductionism of the various Grand Challenges programmes that are said to overlook social, economic, and political contexts (39). Benatar, for example, described the 2008 Institute of Medicine report on the US commitment to global health as regressive, admonishing the organisation for focusing on ‘aspects of health that can be classified medically and treated with medications’ (16). Similarly, Sanders critiques the DALYs approach to estimating the global burden of disease for invariably focusing on medical technologies rather than broader social interventions, such that World Bank recommends oral rehydration salts for diarrhea treatment, rather than basic provision of water and sanitation, which is deemed ‘cost ineffective’ and unsuitable for public sector investment (17).

## The medicalization of global health: three cases

In three subsequent articles I build on these insights, drawing on sociological theories of medicalization to examine three contemporary issues on the current global health agenda – global mental health movement (Paper 2) (40), non-communicable disease agenda (Paper 3) (41), and the universal health coverage campaign (Paper 4) (42). Specifically, I explore areas where these cases might be medicalized: how they are framed as problems in global health and what solutions are presented, to what extent priority issues within these cases became defined as diseases, whether and how strategies for treatment and other health care solutions are offered, what the role of medical providers or of a biomedical model is conceived to be or promoted, and how the role of advocacy groups, civil society, and/or industry reinforce, benefit, or challenge the medicalization of global health. The insights generated offer an assessment as to whether the balance between medical and social considerations are appropriate, why bias might occur, and what the implications are for policy responses and strategies when the framing of global health problems appear too medicalized. If the definitions of global health problems are medicalized, any solutions constructed will similarly be limited in impact. Furthermore, medicalization needs more attention because it has a reproductive or ‘escalating’ aspect that can be harmful: once defined in medical terms (especially by powerful institutions), global health problems and solutions risk being framed as such repeatedly and over time, thus reinforcing the medicalization of global health.

Looking critically at the global health agenda is necessary because it sets priorities and resource allocation, shapes public perceptions and policy decisions, and defines the next generation of hopes and expectations for collective action to save and improve lives. As Kleinman has recently argued (43), more critical reflection on global health problems and programmes using social theories is needed, to complement epidemiological, health services, policy, and ethics studies. And D'Ambruoso specifically in relation to the post-2015 global health agenda notes that: ‘more relevant, inclusive, interdisciplinary analytical frameworks are required to improve notions of what goals are and what success looks like’ (36). Medicalization analyses can provide that needed critical view. While useful critiques have emerged from the many perspectives arguing that the global health agenda overlooks social determinants of health and equity (34–37) and incisive analyses that use social constructionist approaches (44, 45), political economy (17–21), or the ‘right to health’ framework (46, 47) to examine how global health issues get framed or advanced on policy agendas, none to my knowledge have specifically used the lens of medicalization. As a critical sociological tool, a medicalization lens provides a valuable way to uncover some of the limitations and implications of the global health agenda.
